# Glucagon-like peptide-1 receptor agonists and obesity paradox in heart failure with preserved ejection fraction: a systematic review

**DOI:** 10.1097/XCE.0000000000000344

**Published:** 2025-09-17

**Authors:** Darshan Hullon, Karolina Janiec, Violetta Florova, Adam Trach, Yelizaveta Volkova, Ruslan Mnevets

**Affiliations:** a Department of Internal Medicine, MercyOne Clinton Medical Centre, Clinton, Iowa; b Department of Internal Medicine, St. Joseph’s University Medical Center, Paterson, New Jersey; c Department of Internal Medicine, Mount Sinai Hospital Medical Center, Chicago, Illinois, USA; d Department of Internal Medicine, Taras Shevchenko National University of Kyiv; e Institute of Pediatrics, Obstetrics, and Gynecology named after Acad. O.M. Lukyanova of the National Academy of Medical Sciences of Ukraine; f Department of Pediatrics and OBGYN, Taras Shevchenko National University of Kyiv, Kiev, Ukraine

**Keywords:** glucagon-like peptide-1 receptor agonists, heart failure with preserved ejection fraction, obesity

## Abstract

Heart failure with preserved ejection fraction (HFpEF) is associated with obesity, inflammation, and cardiac metabolism. While obesity contributes to HFpEF, the ‘obesity paradox’ suggests that higher BMI may correlate with better outcomes. Glucagon-like peptide-1 receptor agonists (GLP-1 RAs) have cardiovascular benefits through weight loss, anti-inflammatory effects, and improved myocardial function. This systematic review involved randomized trials and cohort studies from 2015 to 2024, assessing GLP-1 RAs in patients with obese HFpEF (BMI ≥ 30 kg/m²). Outcomes included heart failure hospitalizations, mortality, exercise capacity, and quality of life. Eighteen studies involved over 22 000 participants. GLP-1 RAs, especially semaglutide and tirzepatide, consistently reduced weight, inflammation (C-reactive protein), and myocardial stress (N-terminal pro B-type natriuretic peptide) while improving 6-min walk distance and Kansas City Cardiomyopathy Questionnaire scores, uniformly across BMI groups. GLP-1 RAs counter the metabolic burden of obesity in HFpEF while preserving hemodynamic benefits, offering a promising therapeutic option.

## Introduction

Heart failure with preserved ejection fraction (HFpEF) accounts for nearly half of all heart failure cases and involves preserved left ventricular systolic function with impaired diastolic relaxation, which increases filling pressures [1]. Systemic inflammation, endothelial dysfunction, oxidative stress, and cardiac remodeling drive its multifactorial pathophysiology [2–4]. Aging, hypertension, diabetes, and obesity significantly increase the risk of HFpEF [5,6]. The global rise in these conditions and aging populations continues to escalate HFpEF prevalence, surpassing heart failure with reduced ejection fraction (HFrEF) and straining healthcare systems [7].

Clinicians have identified a few effective therapies for HFpEF. Sodium–glucose cotransporter 2 inhibitors, such as empagliflozin and dapagliflozin, reduce cardiovascular mortality and hospitalizations [8,9]. Unlike HFrEF therapies targeting neurohormonal pathways, HFpEF therapies face challenges because of their heterogeneous pathophysiology [10]. Researchers have studied glucagon-like peptide-1 receptor agonists (GLP-1 RAs), including liraglutide, semaglutide, and tirzepatide, which improve glycemic control, promote weight loss, reduce inflammation, and enhance endothelial function in patients with type 2 diabetes [11,12]. Trials like semaglutide treatment effect in people with obesity and HFpEF (STEP-HFpEF) and Study of Tirzepatide Effects on Patients with HFpEF (SUMMIT) demonstrate their benefits in improving symptoms and functional capacity in obesity-associated HFpEF [13,14].

Obesity drives HFpEF through systemic inflammation, epicardial fat deposition, and altered myocardial metabolism [15]; however, studies describe an ‘obesity paradox’, where patients with obese HFpEF experience better outcomes than their normal-weight counterparts [16,17]. Researchers attribute this phenomenon to earlier disease onset, protective adipose tissue properties, and favorable hemodynamic profiles [18]. Weight loss, while reducing obesity-related comorbidities, may negate these survival advantages, particularly if it occurs rapidly or excessively, as shown in studies linking it to worse cardiac outcomes [13,19].

GLP-1 RAs reduce weight selectively while preserving mechanisms associated with potential protection in obese HFpEF subjects. Indeed, they have anti-inflammatory and cardiometabolic effects, which in principle target HFpEF drivers, such as endothelial dysfunction and systemic inflammation [20–22]. This study aimed to summarize the current evidence on the therapeutic actions of GLP-1 RAs in HFpEF, with specific objectives to: (a) evaluate their mechanisms of action and complementary effects to other therapies, (b) assess their impact on clinical outcomes in HFpEF, and (c) examine their interaction with the obesity paradox. By integrating findings from recent mechanistic studies and clinical trials, the study seeks to clarify the role of GLP-1 RAs in managing HFpEF, particularly in obesity-related phenotypes.

## Methodology

We performed this systematic review to assess the efficacy and safety of GLP-1 RAs in the management of the obesity paradox in HFpEF and followed the Preferred Reporting Items for Systematic review and Meta-analysis (PRISMA) guidelines. Eligible studies for inclusion in this review were limited to adults (≥18 years) with a diagnosis of HFpEF (ejection fraction ≥45%) and obesity (BMI ≥ 30 kg/m²) in whom GLP-1 RAs (e.g. semaglutide or liraglutide) were administered. These interventions were compared with placebo, usual care, or alternative treatment options. Main outcomes of interest were major adverse cardiovascular events (MACE), heart failure hospitalizations, and all-cause mortality while secondary outcomes were changes in body weight, BMI, left ventricular ejection fraction, exercise capacity [i.e. 6-min walk distance (6MWD)], quality of life (i.e. Kansas City Cardiomyopathy Questionnaire) and inflammatory bIomarkers; C-reactive protein (CRP) and N-terminal pro B-type natriuretic peptide (NT-proBNP).

Search of major databases, including PubMed, Embase, Cochrane Central Register of Controlled Trials (CENTRAL), and Web of Science were performed to identify relevant studies. Clinical trial registries, such as ClinicalTrials.gov and WHO International Clinical Trials Registry Platform were also searched to identify ongoing or unpublished studies. The search was conducted using a mixture of medical subject headings terms and a variety of free text terms related to HFpEF, GLP-1 RAs, and obesity to ensure a comprehensive and inclusive scope. The search included the period between January 2015 and December 2024, allowing a review of the past 10 years of relevant literature. We included studies published in English from all points of the databases’ inception.

These included randomized controlled trials (RCTs), prospective cohort studies, and case-control studies with predefined eligibility criteria. We excluded narrative reviews, editorials, and retrospective observational studies. Narrative reviews were excluded for the lack of methodological rigor and potential for bias, while retrospective studies were excluded for limitations in causality assessment and susceptibility to bias. All identified studies were screened for eligibility by two independent reviewers after reviewing titles and abstracts. We then assessed full-text articles of possibly in-scope studies for inclusion. A PRISMA flow diagram was used to document the entire selection process.

We extracted data through a standardized, piloted form. Extracted data included study characteristics (author, year, country, design, and degree of funding/economic disclosure); participant demographics (age, sex, race/ethnicity, and baseline BMI and comorbidities); baseline characteristics [HFpEF etiology, New York Heart Association (NYHA) classification, ejection fraction, CRP levels, and NT-proBNP levels]; intervention characteristics (type of GLP-1 RA, dose, duration, and associated medication), comparator details, and reported outcomes. Adverse events and safety data were extracted as well.

Two reviewers independently assessed the risk of bias of the included studies. If the study is an RCT, the Cochrane Risk of Bias 2.0 tool is used, and the Newcastle–Ottawa Scale is used for observational studies. Any disagreements in the risk of bias assessments were resolved by discussion or by consulting a third reviewer.

A narrative synthesis was performed to outline study characteristics, participant characteristics, interventions, and key findings. Measures were summary risk ratios and mean differences with 95% confidence intervals (CIs). Statistical heterogeneity was evaluated by the *I*² statistic and Cochran’s *Q* test. We subsequently performed subgroup analyses based on type of GLP-1 RA, the presence of diabetes, categories of BMI (30–34.9, 35–39.9, and ≥40 kg/m²), and duration of follow-up (2 years). Sensitivity analyses were carried out to explore the robustness of findings and the influence of study quality and possible sources of bias.

## Results

**Fig. 1:**
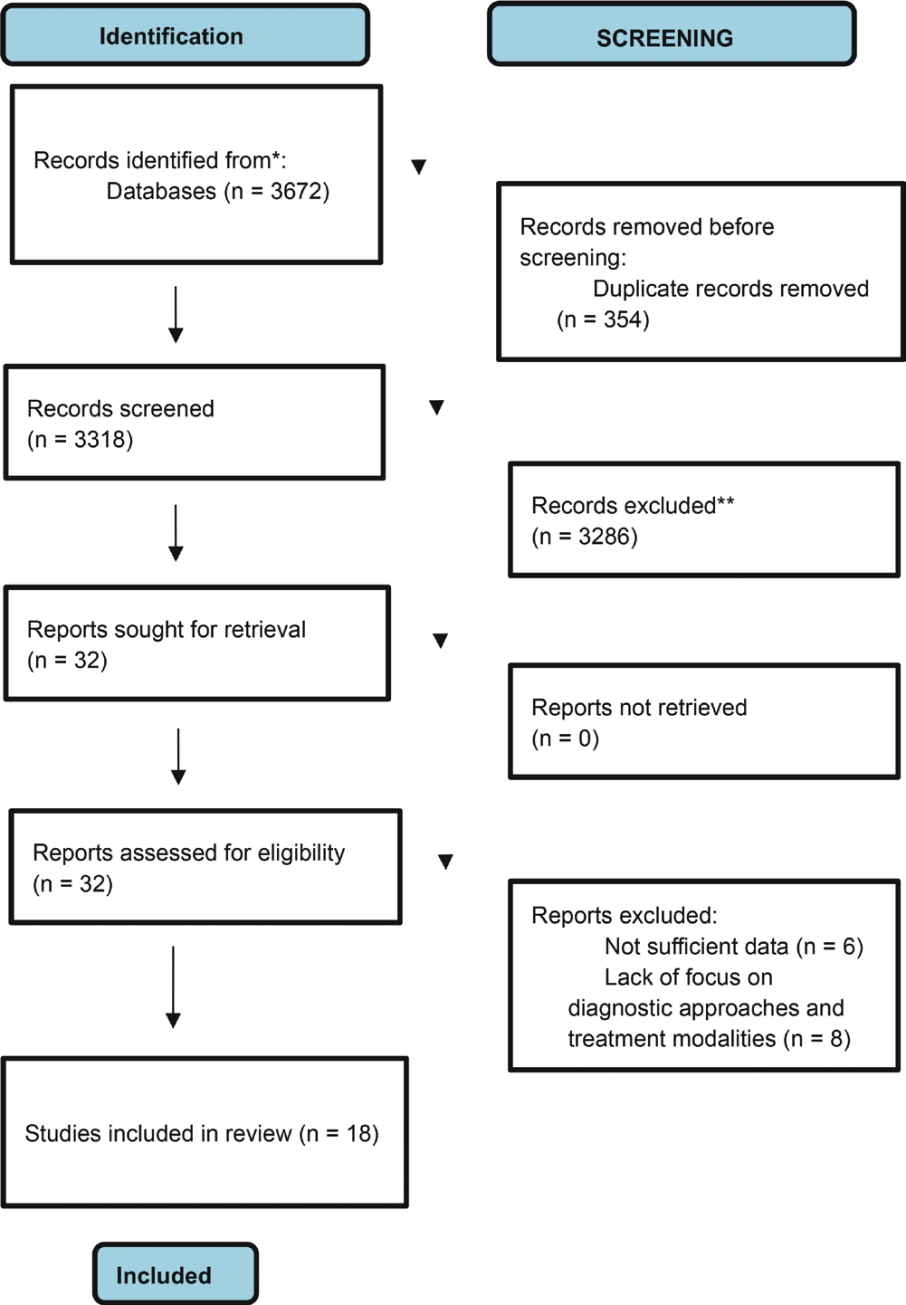
PRISMA 2020 flow diagram.

A total of 3672 studies were identified through the systematic search of electronic databases. After the removal of 354 duplicates, 3318 unique records were screened by title and abstract. Following this initial screening, 32 studies were assessed for eligibility through full-text review. Ultimately, 18 studies met the inclusion criteria and were included in the systematic review. The PRISMA 2020 flow diagram illustrating the study selection process is provided in Fig. 1.

### Study characteristics

This systematic review focused on 18 studies evaluating the implementation of GLP-1 RAs in obesity-related HFpEF, including semaglutide (2.4 mg weekly) and tirzepatide (up to 15 mg weekly). The studies included pooled analysis of RCTs, multinational RCTs, and imaging substudies (*n* ranging from 175 to 22 282) The participants were mostly older adults (median 68–70 years) with BMI greater than or equal to 30 kg/m² and comorbidities like hypertension (73–85%), diabetes (up to 54%), and atrial fibrillation (26–46%). The intervention was administered for 52–104 weeks, and standard HFpEF therapies were provided between groups. The primary outcomes were the composite of Kansas City Cardiomyopathy Questionnaire Clinical Summary Score (KCCQ-CSS), weight loss, decreased heart failure hospitalizations, and decreased mortality. Secondary end points included biomarkers (e.g. NT-proBNP, CRP), exercise capacity (6MWD), and cardiac structural remodeling (e.g. left atrial volume and left ventricular mass). These findings highlight the therapeutic prospects of GLP-1 RAs for the multicomponent pathophysiology of HFpEF. Table 1 shows the study characteristics in detail.

**Table 1:** Study characteristics

References	Design	Population	Intervention	Duration	Primary outcomes	Secondary outcome	Risk of bias
Verma *et al*. [23].	Pooled analysis of STEP-HFpEF and DM trials	1145 (570 women, 575 men)	Semaglutide, 2.4 mg weekly	52 weeks	KCCQ-CSS (+7.6 points), weight loss (−9.6%)	Improvement in 6MWD, reduction in CRP	Low
Butler *et al*. [24].	Pooled analysis of two RCTs	1145	Semaglutide, 2.4 mg weekly	52 weeks	KCCQ-CSS (+7.5 points), weight loss (−8.4%), HF hospitalization (HR: 0.27)	Increased 6MWD (+17.1 m), reduced CRP (−36%), NT-proBNP (−18%),	Low
Petrie *et al*. [25].	Pooled analysis of two RCTs	1145	Semaglutide, 2.4 mg weekly	52 weeks	NT-proBNP reduction (−22%), KCCQ-CSS (+7.5 points), weight loss (−8.4%)	Improvement in 6MWD (+17.1 m), reduction in CRP	Low
Schou *et al*. [14].	Pooled analysis of two RCTs	1145	Semaglutide, 2.4 mg weekly	52 weeks	NYHA improvement (32.6%), KCCQ-CSS (+7.5 points), HF hospitalizations (HR: 0.36)	Improved 6MWD (+17.1 m overall), NT-proBNP (−18%), CRP (−36%)	Low
Solomon *et al*. [8].	Echocardiographic substudy	491	Semaglutide, 2.4 mg weekly	52 weeks	LA volume reduction (−6.13 ml), improved diastolic function	Improved diastolic function (E/e′ reduced by −0.79; *P* = 0.05), better E/A ratio (−0.14; *P* = 0.0075).	Low
Verma *et al*. [26].	Pooled analysis of STEP-HFpEF trials	1145 (518 with AF and 627 without AF)	Semaglutide, 2.4 mg weekly	52 weeks	KCCQ-CSS improvement (+11.5 points in AF), NT-proBNP reduction (−22%)	6MWD (+17.1 m), CRP reduction (−36%)	Low
Verma *et al*. [27].	Pooled analysis of two RCTs	1145	Semaglutide, 2.4 mg weekly	52 weeks	KCCQ-CSS improvement (+7.5 points overall), weight loss (−8.4%)	6MWD (+17.1 m), CRP reduction (−36%), NT-proBNP reduction (−22%)	Low
Kosiborod *et al*. [28].	Pooled analysis of STEP-HFpEF and DM trials	1146	Semaglutide, 2.4 mg weekly	52 weeks	KCCQ-CSS improvement (+7.5 points overall), weight loss (−8.4%)	6MWD (+17.1 m), NT-proBNP (-22%), CRP (−36%)	Low
Shah *et al*. [29].	Pooled analysis of two RCTs	1145	Semaglutide, 2.4 mg weekly	52 weeks	KCCQ-CSS (+7.5 points), weight loss (−8.4%), loop diuretic reduction (−17%)	6MWD (+17.1 m), CRP reduction (−36%), NT-proBNP (−22%)	Low
Kosiborod *et al*. [19].	Multinational RCT	529	Semaglutide, 2.4 mg weekly	52 weeks	KCCQ-CSS (+7.8 points), weight loss (−10.7%), NT-proBNP (−20.9%)	6MWD improvement (+20.3 m; *P* < 0.001), CRP reduction (−43.5%; *P* < 0.001)	Low
Kosiborod *et al*. [30].	Multinational RCT (STEP-HFpEF DM)	616	Semaglutide, 2.4 mg weekly	52 weeks	KCCQ-CSS (+7.3 points), weight loss (−9.8%), NT-proBNP reduction (−23.2%)	6MWD (+14.3 m, *P* = 0.008), CRP reduction (−42%, *P* < 0.001)	Low
Kosiborod *et al*. [31].	Randomized, double-blind, placebo-controlled trial (STEP-HFpEF)	529	Semaglutide, 2.4 mg weekly	52 weeks	KCCQ-CSS (+7.8 points), weight loss (−11%)	6MWD (+20 m), CRP reduction, and NT-proBNP levels	Low
Deanfield *et al*. [32].	Multinational RCT	17 604 (4286 with HF)	Semaglutide, 2.4 mg weekly	~40 months	MACE (HR: 0.72), HF hospitalization (HR: 0.79), CV death (HR: 0.76)	Reductions in HF hospitalizations and symptom improvement in HFpEF subgroup	Low
Kosiborod *et al*. [33].	Pooled analysis of RCTs	22 282 (3743 with HFpEF)	Semaglutide, 2.4 mg weekly	52 weeks	CV death or HF hospitalization (HR: 0.69)	Worsening HF events (HR: 0.59), serious adverse events (29.9%)	Low
Borlaug *et al*. [13].	Multinational RCT (SUMMIT)	731	Tirzepatide, up to 15 mg weekly	52 weeks	Reduction in systolic BP (−5 mmHg), weight loss (−11.6%), CRP (−37.2%)	Improved 6MWD (+18.3 m), decreased troponin T (−10.4%), reduced NT-proBNP (−10.5%)	Low
Zile *et al*. [34].	Multinational RCT	731	Tirzepatide, up to 15 mg weekly	~104 weeks	CV death or worsening HF (HR: 0.62), KCCQ-CSS improvement (+6.9 points)	Weight loss: (−11.6%), exercise capacity: 6MWD improvement (+18.3 m), CRP reduction (−37.2%)	Low
Packer *et al*. [35].	Multinational RCT	731	Tirzepatide, up to 15 mg weekly	~104 weeks	CV death or worsening HF (HR: 0.62), weight loss (−11.6%), KCCQ-CSS improvement: (+6.9 points)	Improved 6MWD (+18.3 m), CRP reduction (−34.9%)	Low
Kramer *et al*. [36].	CMR substudy of SUMMIT	175	Tirzepatide, up to 15 mg weekly	52 weeks	LV mass (−11 g), paracardiac adipose (−45 ml)	LV end-diastolic volume reduction: (−7 ml)	Low

BP, blood pressure; CMR, cardiac magnetic resonance; CRP, C-reactive protein; CV, cardiovascular; DM, diabetes mellitus; HF, heart failure; HFpEF, heart failure with preserved ejection fraction; HR, hazard ratio; KCCQ-CSS, Kansas City Cardiomyopathy Questionnaire Clinical Summary Score; LA, left atrial; LV, left ventricular; MACE, major adverse cardiovascular events; 6MWD, 6-min walk distance; NT-proBNP, N-terminal pro B-type natriuretic peptide; NYHA, New York Heart Association; RCT, randomized controlled trial; STEP-HFpEF, semaglutide treatment effect in people with obesity and HFpEF; SUMMIT, Study of Tirzepatide Effects on Patients with HFpEF.

### Mechanisms of action

#### Anti-inflammatory effects

GLP-1 RAs had a consistent effect on lowering systemic inflammation, with each of the studies reporting a significant decrease in CRP levels. CRP was reduced by 36–42% following semaglutide (*P* < 0.001), including a 36% reduction in the STEP-HFpEF program and a greater 42% reduction in pooled analyses of the STEP-HFpEF studies [27,33]. Similar reductions of 34.9–37.2% were observed in the SUMMIT and other trials with tirzepatide [13,34,35]. These reductions were correlated with better functional and quality-of-life measures (e.g. KCCQ-CSS, exercise capacity) (Table 2).

**Table 2:** Anti-inflammatory effects of glucagon-like peptide-1 receptor agonist

Study title	Intervention	CRP reduction (%)	*P* value	References
STEP-HFpEF program	Semaglutide, 2.4 mg weekly	36	<0.001	Verma *et al*. [26,27]
STEP-HFpEF DM trial	Semaglutide, 2.4 mg weekly	42	<0.001	Kosiborod *et al*. [30]
SUMMIT trial	Tirzepatide, up to 15 mg	37.2	<0.001	Borlaug *et al*. [13]
SUMMIT CMR substudy	Tirzepatide, up to 15 mg	34.9	<0.001	Packer *et al*. [35]

CMR, cardiac magnetic resonance; CRP, C-reactive protein; DM, diabetes mellitus; HFpEF, heart failure with preserved ejection fraction; STEP-HFpEF, semaglutide treatment effect in people with obesity and HFpEF; SUMMIT, Study of Tirzepatide Effects on Patients with HFpEF.

#### Endothelial function and myocardial remodeling

##### N-terminal pro B-type natriuretic peptide reductions

Semaglutide reduced NT-proBNP by 18–23% across studies, indicating reduced myocardial wall stress. The STEP-HFpEF trial demonstrated a 22% NT-proBNP reduction with semaglutide compared to placebo, highlighting significant improvements in myocardial stress markers [25]. Pooled analyses of RCTs, including STEP-HFpEF, reported NT-proBNP reductions averaging 20% with semaglutide treatment [24]. Tirzepatide also significantly reduced NT-proBNP by 20.9%, as shown in the SUMMIT trial, indicating similar efficacy in reducing myocardial stress [13] (Table 3).

**Table 3:** N-terminal pro B-type natriuretic peptide reduction across studies

Study title	Intervention	NT-proBNP reduction (%)	*P* value	References
STEP-HFpEF	Semaglutide, 2.4 mg weekly	18	<0.001	Butler *et al*. [24].
NT-proBNP insights	Semaglutide, 2.4 mg weekly	22	<0.001	Petrie *et al*. [25].
SUMMIT trial	Tirzepatide, up to 15 mg	20.9	<0.001	Borlaug *et al*. [13].

HFpEF, heart failure with preserved ejection fraction; NT-proBNP, N-terminal pro B-type natriuretic peptide; STEP-HFpEF, semaglutide treatment effect in people with obesity and HFpEF; SUMMIT, Study of Tirzepatide Effects on Patients with HFpEF.

##### Cardiac structural and functional changes

Semaglutide reduced left atrial volume by 6.13 ml in an echocardiographic substudy of the STEP-HFpEF trial [8] (*P* = 0.0013). In addition, diastolic function improved significantly, as measured by E/e′ ratios (*P* = 0.05). Tirzepatide demonstrated structural benefits, reducing left ventricular mass by 11 g and paracardiac adipose tissue by 45 ml in the SUMMIT CMR substudy [36] (*P* < 0.004). These structural changes directly reflect improvements in myocardial remodeling (Table 4).

**Table 4:** Cardiac structural and functional improvements

Study title	Intervention	LA volume change (ml)	LV mass change (g)	*P* value	References
Echocardiographic substudy (STEP-HFpEF)	Semaglutide, 2.4 mg weekly	−6.13	N/A	0.0013	Solomon *et al*. [8].
SUMMIT CMR substudy	Tirzepatide, up to 15 mg	N/A	−11	0.004	Kramer *et al*. [36].

CMR, cardiac magnetic resonance; HFpEF, heart failure with preserved ejection fraction; LA, left atrial; LV, left ventricular; STEP-HFpEF, semaglutide treatment effect in people with obesity and HFpEF; SUMMIT, Study of Tirzepatide Effects on Patients with HFpEF.

### Clinical outcomes

#### Cardiovascular mortality and hospitalizations

GLP-1 RAs, including semaglutide and tirzepatide, consistently demonstrated reductions in heart failure hospitalizations and cardiovascular mortality across trials. In the STEP-HFpEF trial [24], semaglutide reduced heart failure hospitalization risk with a hazard ratio of 0.27 over 52 weeks. Similarly, the SELECT trial [31] reported significant reductions in heart failure hospitalization rates (hazard ratio: 0.79) and MACE with semaglutide (hazard ratio: 0.72, 95% CI: 0.60–0.87) over approximately 40 months. In the SUMMIT trial [13], tirzepatide significantly reduced the risk of cardiovascular death or worsening heart failure with an hazard ratio of 0.62 (95% CI: 0.41–0.95, *P* = 0.026). A pooled analysis of the STEP trials [14] further confirmed significant reductions in heart failure hospitalizations, reporting an hazard ratio of 0.36 (Table 5).

**Table 5:** Clinical outcomes

Outcome	Semaglutide (STEP-HFpEF)	Tirzepatide (SUMMIT)	Comments
HF hospitalization	HR: 0.27–0.36	HR: 0.62 (95% CI: 0.41–0.95)	Both drugs showed significant reductions in HF hospitalization risk
Cardiovascular death or MACE	HR: 0.72 (95% CI: 0.60–0.87)	HR: 0.62	Semaglutide and tirzepatide reduced MACE and CV deaths consistently
6MWD improvement	+17.1 to +20.3 m	+18.3 m (95% CI: 9.9–26.7)	Improvements in exercise capacity were clinically meaningful across all trials
KCCQ-CSS	+7.5 to +11.9 points	+6.9 points	Consistent symptom relief exceeding clinical thresholds
Weight loss	−8.4 to −10.7%	−11.6%	Weight loss correlated with reductions in cardiac remodeling and symptoms

HFpEF, heart failure with preserved ejection fraction; CI, confidence interval; CV, cardiovascular; HF, heart failure; HR, hazard ratio; KCCQ-CSS, Kansas City Cardiomyopathy Questionnaire Clinical Summary Score; MACE, major adverse cardiovascular events; 6MWD, 6-min walk distance; STEP-HFpEF, semaglutide treatment effect in people with obesity and HFpEF; SUMMIT, Study of Tirzepatide Effects on Patients with HFpEF.

#### Exercise capacity and symptoms

Both semaglutide and tirzepatide demonstrated substantial improvements in exercise capacity and heart failure–related symptoms. Improvements in the 6MWD were consistently observed across trials. The STEP-HFpEF trials reported an average improvement of +17.1 m [25]. Similarly, the SUMMIT trial [13] showed an improvement of +18.3 m (95% CI: 9.9–26.7, *P* < 0.001) with tirzepatide. Kosiborod *et al*. [19,30,31,33] highlighted greater gains in patients with elevated NT-proBNP tertiles, with improvements reaching +20.3 m (Table 5).

Symptom relief, measured by the Kansas City Cardiomyopathy Questionnaire Clinical Summary Score (KCCQ-CSS), consistently exceeded thresholds for clinical significance across trials. Mean improvements ranged from +6.9 to +11.9 points. In the STEP-HFpEF trial [25], semaglutide improved KCCQ-CSS by +7.5 points overall, with a more pronounced improvement of +11.5 points in patients with atrial fibrillation. The SUMMIT trial reported a KCCQ-CSS improvement of +6.9 points (95% CI: 3.3–10.6, *P* < 0.001) with tirzepatide.

#### Quality of life and weight loss

GLP-1 RAs significantly improved quality of life and induced meaningful weight loss, which correlated with better cardiovascular outcomes. In the SUMMIT trial, tirzepatide achieved a weight reduction of −11.6% (95% CI: −12.9 to −10.4%, *P* < 0.001), alongside reductions in paracardiac adipose tissue and improvements in diastolic function. In the pooled analysis of the STEP-HFpEF trials, semaglutide resulted in a weight loss ranging from −8.4 to −10.7%, with these reductions correlating with improved exercise capacity and significant decreases in left atrial volume (Table 5).

Improvements in cardiac function were also observed. The STEP-HFpEF echocardiographic substudy [8] reported a reduction in left atrial volume by −6.13 ml (*P* = 0.0013) with semaglutide, alongside enhanced diastolic function as indicated by a reduction in E/e′ by −0.79.

### Obesity paradox interaction

GLP-1 RAs, such as semaglutide and tirzepatide, demonstrated consistent benefits across both obese and nonobese populations while addressing concerns about rapid or excessive weight loss, which could undermine the hemodynamic and survival advantages linked to obesity. Subgroup analyses confirmed the efficacy of GLP-1 RAs across different BMI categories, with improvements in cardiovascular outcomes, quality of life, and exercise capacity being comparable in patients with obese and nonobese HFpEF. For instance, the STEP-HFpEF trials reported a significant improvement of +7.5 points in KCCQ-CSS (quality-of-life scores), with no interaction between BMI subgroups. Weight loss ranged from 8.4 to 11.6%, yielding proportionate benefits for all participants (Table 6). Despite inducing weight reduction, GLP-1 RAs preserved the hemodynamic advantages of obesity by mitigating related comorbidities like hypertension and diabetes without compromising survival benefits. Reductions in NT-proBNP and CRP biomarkers across all BMI groups highlighted sustained improvements in myocardial and endothelial function. In addition, trials ensured gradual weight loss through dose titration protocols, with an average reduction of 8.4–10.7% over 52 weeks, preserving the favorable hemodynamic profiles of obesity-associated HFpEF. Improvements in exercise capacity, as measured by a 6MWD increase of up to +20.3 m, and in NYHA functional class were consistent across BMI subgroups, with obese patients retaining their functional advantage in exercise tolerance. These findings underscore the uniform efficacy of GLP-1 RAs in addressing HFpEF’s complex pathophysiology while maintaining the benefits of the obesity paradox (Table 7).

**Table 6:** Subgroup effects of glucagon-like peptide-1 receptor agonist on quality of life and weight loss

Study	Population (BMI)	KCCQ-CSS improvement	Weight loss (%)
STEP-HFpEF [25]	BMI ≥ 30 kg/m² (obese)	+7.6	−9.6
STEP-HFpEF [23]	BMI < 30 kg/m² (nonobese)	+7.5	−8.4
SUMMIT [36]	Mean BMI 38.3 kg/m²	+6.9	−11.6

HFpEF, heart failure with preserved ejection fraction; KCCQ-CSS, Kansas City Cardiomyopathy Questionnaire Clinical Summary Score; STEP-HFpEF, semaglutide treatment effect in people with obesity and HFpEF; SUMMIT, Study of Tirzepatide Effects on Patients with HFpEF.

**Table 7:** Impact of glucagon-like peptide-1 receptor agonists on biomarkers and hospitalizations (BMI subgroups)

Study	BMI subgroup	CRP reduction (%)	NT-proBNP reduction (%)	HF hospitalization HR
STEP-HFpEF [24]	BMI ≥ 30 kg/m²	−36	−22	0.27
STEP-HFpEF [14]	BMI < 30 kg/m²	−36	−22	0.36
SUMMIT [13]	All BMI	−37.2	−23	0.62

CRP, C-reactive protein; HF, heart failure; HFpEF, heart failure with preserved ejection fraction; HR, hazard ratio; KCCQ-CSS, Kansas City Cardiomyopathy Questionnaire Clinical Summary Score; STEP-HFpEF, semaglutide treatment effect in people with obesity and HFpEF; SUMMIT, Study of Tirzepatide Effects on Patients with HFpEF.

### Heterogeneity in glucagon-like peptide-1 receptor agonists

Heterogeneity across studies stemmed from variations in sample sizes, comorbidities, and HFpEF definitions (Table 8). Sample sizes ranged from 175 to 22 282 participants, with differing prevalences of hypertension (73–85%), diabetes (up to 54%), and atrial fibrillation (26–46%). HFpEF definitions varied, using either LVEF thresholds (≥45%) or NT-proBNP levels. Subgroup analyses showed consistent NT-proBNP reductions (18–23%) and quality-of-life improvements (KCCQ-CSS + 7 points) across BMI and NT-proBNP tertiles.

**Table 8:** Key differences between semaglutide and tirzepatide trials

Aspect	Semaglutide	Tirzepatide
Sample size	491–17 604 participants	175–731 participants
Weight loss (%)	8.4–10.7%	11.6% (95% CI: 10.4–12.9%)
Cardiac structure	Limited improvements in LA volume	Significant LV mass reduction (−11g)
Biomarker changes	NT-proBNP reduction: ~22%	NT-proBNP reduction: ~23%
Trial duration	52 weeks to ~40 months	52 to ~104 weeks

CI, confidence interval; LA, left atrial; NT-proBNP, N-terminal pro B-type natriuretic peptide.

Tirzepatide trials reported larger weight loss (up to 11.6%) and greater cardiac improvements (e.g. left ventricular mass reduction: −11 g) compared with semaglutide, likely because of its dual GLP-1/GIP mechanism. Despite heterogeneity, GLP-1 RAs demonstrated uniform efficacy across populations, supporting their broad applicability in HFpEF management.

## Discussion

### The obesity paradox: the U-shaped relationship

The ‘obesity paradox’ is an observable trend in patients with heart failure, where those with higher BMI often experience improved survival benefits [16,37]. There are multiple published studies cognizant of a U-shaped relationship between BMI and mortality, in which the risk of mortality is increased at both extremes of BMI (low and high), while mid-range BMI appears to be cardioprotective [38,39]. This finding contrasts with the classic notion of obesity as a risk factor for cardiovascular diseases because obesity contributes to systemic inflammation, epicardial fat accumulation, and changes in myocardial metabolism, all promoters of HFpEF [37,40]. This paradoxical observation has sparked extensive discussion in the medical community.

### Evidence supporting the obesity paradox in heart failure with preserved ejection fraction

The supporting evidence for the obesity paradox in HFpEF includes TOPCAT and ARISTOTLE. In these two large cohort studies, patients with obese HFpEF had mortality and hospitalization rates lower than their normal-weight counterparts [16,41]. The observation of these findings suggested obesity may impart a protective effect in specific cardiovascular disease rather being a risk factor. Additional observational studies reported improved cardiac output, more favorable hemodynamic profiles, and greater metabolic reserves in higher patients with BMI [16].

The beneficial effects in patients with obese HFpEF may be mechanistically related to multiple factors. For instance, adipose tissue serves as an endocrine organ that secretes factors, including anti-inflammatory adipokines, which attenuate systemic inflammation [42,43]. This effect could counteract inflammation processes typical of HFpEF. In addition, obese patients may present with elevated circulating lipoproteins, which may afford some buffering against cardiovascular insults [16]. In addition, increased energy storage in the form of fat tissue in obese patients can be especially beneficial in these acute decompensating episodes, creating essentially a metabolic buffer that improves survival rates [37].

Despite these findings of higher BMI and better outcomes, they have not been consistently reproduced in all studies. This lack of reproducibility may suggest methodological bias underlying this paradox [44].

### Counter evidence to the obesity paradox

The obesity paradox suggests increased weight is associated with improved survival with an adage that decreasing weight may increase mortality according to the U-shaped relationship. Recent evidence counters this paradox in the context of HFpEF when treated with GLP-1 RAs. In the SELECT trial, weight loss improves cardiovascular outcomes, in particular decreasing cardiovascular mortality [45]. This finding argues against that lower BMI may increase mortality.

In addition, the STEP-HfpEF trial challenged the paradigm that weight loss impairs physiological reserve. This trial showed hardware gains in functional capacity with improved exercise tolerance as seen in 5 min walk test and a significant reduction in CRP and NT-proBNP [46]. These findings underscore the promise of weight loss strategies to treat the underlying pathophysiology of HfpEF rather than worsening.

Another challenge to the obesity paradox is biological plausibility. Although high BMI is correlated with certain compensatory mechanisms, it often conceals the detriment of central adiposity and ectopic fat deposition, contributing to heightened cardiovascular risk. Studies, such as advanced imaging studies, have established an eminent role of visceral fat accumulation in driving myocardial dysfunction and systemic inflammation and thus compromising the observed benefits of obesity [47]. Finally, the use of BMI as a measure of obesity ignores fat distribution, muscle mass, and other important determinants of health outcomes [48]

Overall, these results suggest that the obesity paradox is more of a statistical artifact than a true physiological phenomenon. There are also methodological biases, such as reverse causality and collider stratification bias, that question its validity as they generate false associations between obesity and survival [16,49].

### Future directions of research

A multidimensional research strategy is needed to address the debate over the obesity paradox since heterogeneity exists. First, there is an urgent need for large-scale meta-analyses combining data from GLP-1 RA assays and other weight loss interventions carried out in patients with HFpEF. A recent meta-analysis of nine RCTs, including 8920 patients with heart failure and type 2 diabetes, reported a 13% reduction in MACEs with GLP-1 RA treatment compared with placebo [50]; however, this analysis did not differentiate between heart failure phenotypes, highlighting the need for more nuanced investigations specifically focused on HFpEF. To ensure the robustness of findings, advanced statistical techniques should be employed to account for potential confounders and biases.

Second, the body roundness index (BRI) and a body shape index (ABSI) have shown promise in assessing obesity-related health risks. A study by Christakoudi *et al*. [51] found that ABSI was a better predictor of metabolic syndrome than traditional measures like BMI in some populations; however, conflicting results have been reported, with some studies suggesting that ABSI and BRI are poor predictors of metabolic syndrome risk factors in overweight and obese individuals [52]. This inconsistency underscores the need for further research to clarify the utility of these indices in HFpEF populations.

Third, future and ongoing clinical trials may further clarify the relationship between weight loss and HFpEF events. Such trials, NCT05878912 and NCT05942287 [53,54], will generate important data on the benefit of GLP-1 RAs (and potentially) other interventions in obesity-related outputs unique to HFpEF populations. Such studies should also explore the effects of these interventions on relevant subgroups, including those defined according to sex, age, or baseline metabolic status, to identify those populations that might derive maximal benefit from individual therapies.

Last, mechanistic studies are needed to investigate the molecular signaling pathways by which obesity and weight loss modulate HFpEF progression. Research into the molecular pathways linking obesity, weight loss, and HFpEF progression is ongoing. Studies have shown that GLP-1 RAs may exert cardioprotective effects through multiple mechanisms, including reducing epicardial fat tissue thickness, improving myocardial energy metabolism, and reducing systemic inflammation [20,21]. Furthermore, investigations into the role of epicardial adipose tissue in HFpEF have revealed its potential as a therapeutic target. A study by Jalil *et al*. [55] demonstrated that liraglutide treatment significantly reduced epicardial fat thickness in patients with type 2 diabetes. The implications of these findings would benefit from further multidisciplinary partnerships across academia and the Ministry of Health.

### Conclusion

GLP-1 RAs utilize a multimechanistic approach to target not only hyperglycemia but also obesity and cardiovascular risk factors, which is contradictory to the rationale of the obesity paradox. These findings further direct that this observational paradox fails to be a true physiological phenomenon and possibly a statistical and methodological artifact. In this context, these agents represent a paradigm not only for a different class of drugs for the management of HFpEF, but also highlight the importance of investigating obesity as a modifiable risk factor for HFpEF. Ultimately, such debate and determining the appropriate therapeutic paradigm for patients with HFpEF will be most conclusively settled by emerging evidence driven by next-generation metrics and clinical trial designs.

## Acknowledgements

The authors thank the contributors of the referenced studies and the teams responsible for trial designs and publications that made this systematic review possible.

Conceptualized the study, supervised the study, and provided critical revisions: D.H. Conducted the literature search, performed risk of bias assessments, reviewed statistical methodologies, and performed data analysis: A.T., Y.V., and R.M. Contributed to result interpretation, drafted the manuscript, and contributed to manuscript editing: D.H., K.J., and V.F. All authors read and approved the final manuscript.

The datasets generated and/or analyzed during the current study are derived from published articles and publicly available clinical trial databases. Specific references and sources are cited within the manuscript.

### Conflicts of interest

There are no conflicts of interest.
